# Purtscher-Like Retinopathy and Diffuse Alveolar Hemorrhage Caused by Soft Tissue Injection for Gluteal Augmentation

**DOI:** 10.4172/2155-9570.1000402

**Published:** 2015-02-27

**Authors:** Laura C. Huang, Basil K Williams, Audrey C Ko, Zohar Yehoshua, Chrisfouad R Alabiad

**Affiliations:** Bascom Palmer Eye Institute, University of Miami, Miller School of Medicine, Miami, FL, USA

**Keywords:** Cosmetic filler injection, Gluteal augmentation, Purtscher retinopathy, Purtscher-like retinopathy, Soft tissue filler

## Abstract

**Purpose:**

To describe a rare occurrence of acute vision loss and diffuse alveolar hemorrhage following a treatment of injectable gluteal cosmetic filler.

**Patient and methods:**

A 20-year-old female underwent a cosmetic injection of unknown components for gluteal augmentation. Within hours she developed progressive shortness of breath secondary to diffuse alveolar hemorrhage. She presented to ophthalmology 6 weeks later with a history of bilateral decreased vision. Clinical examination revealed cotton wool spots and retinal hemorrhages. Fluorescein angiography demonstrated macular vascular pruning and an enlarged foveal avascular zone.

**Results:**

The patient was observed and vision did not improve after 8 months of follow-up.

**Conclusion:**

These findings were attributed to a Purtscher-like retinopathy secondary to systemic inflammation induced by the filler and/or direct microembolization of the injected material or fat. To the best of the authors’ knowledge, this is the first documented case of diffuse alveolar hemorrhage and ischemic bilateral vision loss in a patient undergoing gluteal augmentation with dermal filler.

## Introduction

Minimally invasive cosmetic procedures are being performed with increasing frequency as a means to circumvent surgery. Soft tissue filler injections are a frequently performed non-invasive technique, second only to botulinum toxin [1]. Despite the convenience and simplicity of these office-based procedures, complications are possible, particularly when performed by inexperienced or unlicensed individuals. Ocular complications of dermal fillers are rare, but can occur secondary to retrograde flow in the ophthalmic artery [2,3]. This is the first reported case of acute bilateral vision loss and diffuse alveolar hemorrhage in the setting of cosmetic filler injection for gluteal augmentation.

## Case Report

A 20-year-old female received an injection of approximately 150 cm^3^ of what she described as “Venezuelan Artefill” for gluteal augmentation 6 weeks prior to presentation. The procedure was performed by an unlicensed administrator in a massage parlor. Immediately after the injection, she developed acute shortness of breath that was treated as an asthma exacerbation in the emergency room. She was admitted for persistent shortness of breath 2 days later. She developed frank hemoptysis and oxygen dependence. Computed tomography demonstrated the development of ground glass opacities of her lungs. Bronchoscopy and alveolar lavage confirmed a diagnosis of diffuse alveolar hemorrhage. Concurrently, the patient suffered a precipitous drop in her hematocrit (35% to 20%) over the course of 48 hours. She was ultimately stabilized with adequate hydration, high dose steroids, and two blood transfusions.

**Figure 1:**
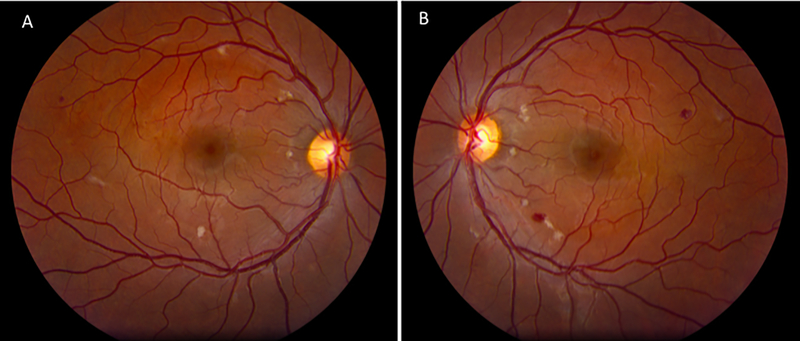
Fundus photography at time of presentation. A: Fundus photograph of the right eye at presentation depicting cotton wool spots and intraretinal hemorrhages localized to the posterior pole. B: Fundus photograph of the left eye at presentation depicting a similar presentation.

After discharge, she presented to the ophthalmology clinic with a history of acute bilateral vision loss that began the day of the cosmetic injection. The best-corrected visual acuity was 20/70 in the right eye and 20/200 in the left eye. Dilated fundoscopic examination revealed multiple cotton wool spots and intraretinal hemorrhages (Figure 1).

**Figure 2:**
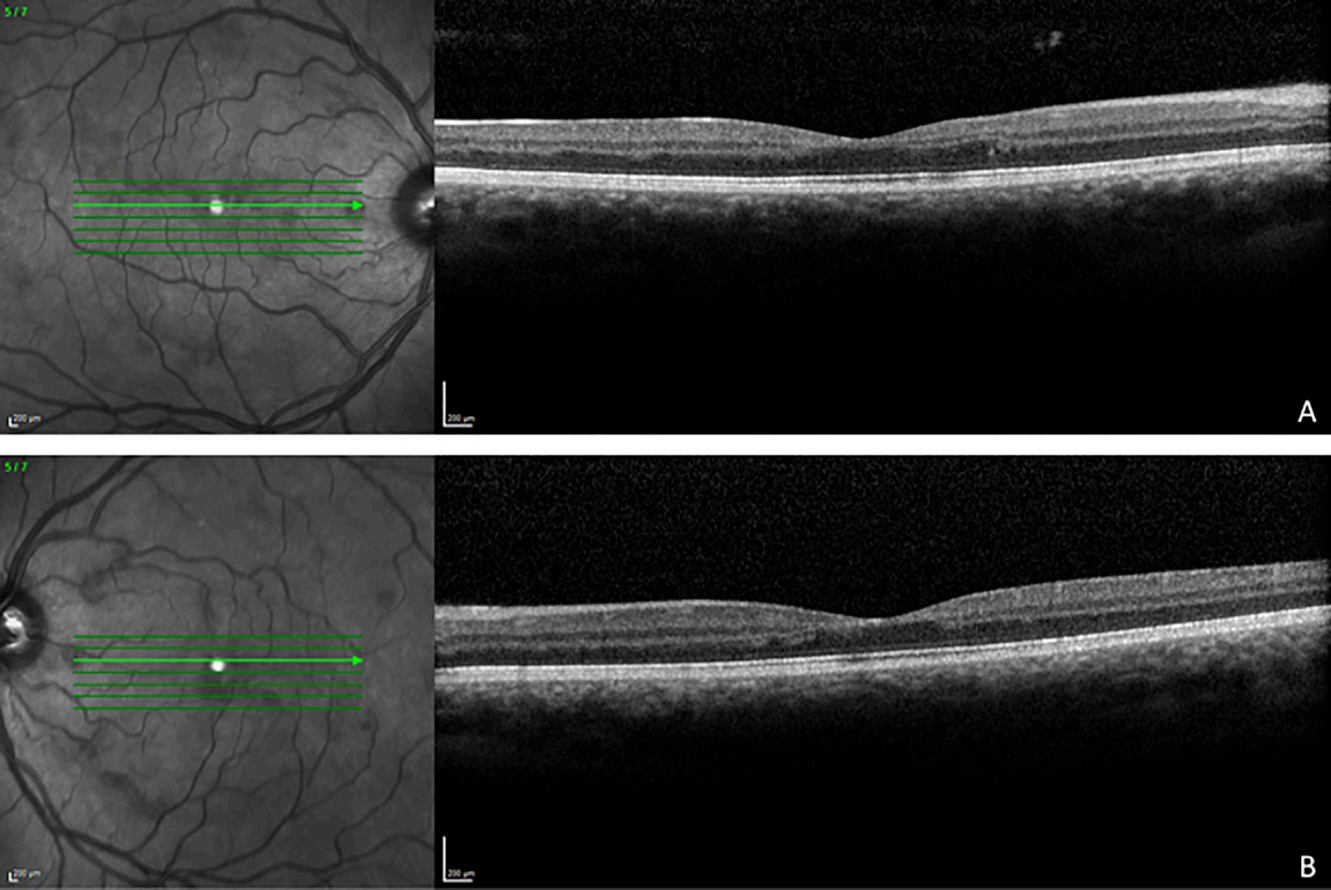
Optical coherence tomography at time of presentation. A: Spectral-domain optical coherence tomography scan of the right eye at presentation demonstrating gross irregularity of the inner and outer retina with partial loss of the outer plexiform layer. B: Spectral-domain optical coherence tomography scan of the left eye at presentation demonstrating an analogous appearance.

**Figure 3:**
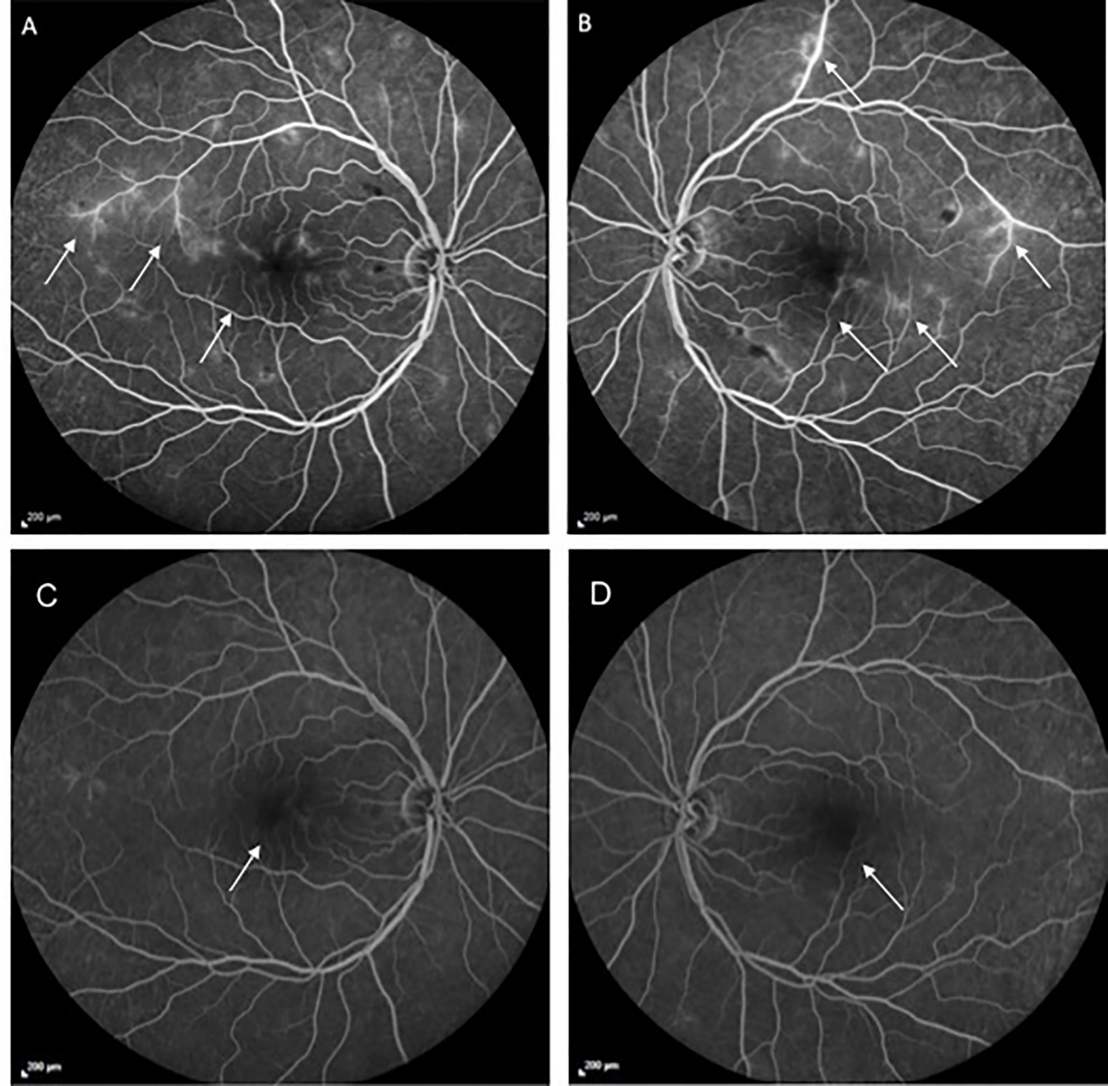
Late phase fluorescein angiogram at time of presentation and at most recent follow-up. A: Late phase fluorescein angiogram of the right eye at presentation showing blockage from the retinal hemorrhages, diffuse vascular leakage, temporal capillary non-perfusion, and an enlarged foveal avascular zone. B: Late phase fluorescein angiogram of the left eye at presentation showing a comparable appearance. C: Late phase fluorescein angiogram of the right eye at most recent follow-up indicating a resolution of retinal hemorrhages and significant reduction in the vascular leakage. The temporal capillary non-perfusion and the enlarged foveal avascular zone persist. D: Late phase fluorescein angiogram of the left eye at most recent follow-up indicating a similar progression.

Spectral-domain ocular coherence tomography (Spectralis, Heidelberg Engineering, Vista, CA) of the macula demonstrated irregularities in both the inner and outer retina without evidence of retinal edema (Figure 2). Fluorescein angiography (Visupac, Carl Zeiss Meditec, Dublin, CA, USA) displayed diffuse macular leakage and vascular pruning causing an enlarged Foveal Avascular Zone (FAZ) (Figure 3).

Observation was recommended. After 8 months of follow-up, the cotton wool spots and intraretinal hemorrhages resolved. However, the visual acuity further deteriorated to 20/200 in the right eye and 20/400 in the left eye, and the enlarged FAZ remained on fluorescein angiography (Figure 3). Continued observation was recommended.

## Discussion

Cosmetic injections by untrained practitioners in nonclinical settings have been performed for over a century. Local complications are well-documented and include granulomatous inflammatory reactions, erythema, itching, depigmentation, induration, ulceration, and tissue necrosis [4]. Vision loss secondary to retrograde embolization after cosmetic facial filler injection has also been described as a local complication [2,3]. Systemic complications are less frequent, and acute vision loss from non-facial aesthetic injections has not been documented previously.

To the authors’ knowledge, this is the first reported case of soft tissue filler injection for gluteal enhancement leading to bilateral vision loss and diffuse alveolar hemorrhage. The specific preparation of material injected in this case remains unknown. At the time of administration, the patient was told she was receiving a variation of polymethylmethacrylate (PMMA) and bovine collagen mixed with an unspecified additional ingredient and labeled as “Venezuelan Artefill” because she could not afford the pure product. Artefill^®^ (Suneva Medical, San Diego, CA) is a third-generation PMMA product that is FDA approved for the treatment of nasolabial folds, but is often used off-label for other indications [5]. It is important to note that it is unknown if Artefill was used as a component of the treatment, and that neither of these complications have been previously reported with the use of Artefill.

Multiple pulmonary vascular effects, including diffuse alveolar hemorrhage, have been described with subcutaneous cosmetic injections of various unconventional viscous substances including castor and corn oil, petroleum jelly, and automobile transmission fluid [6–8]. The most commonly used illicit substance is liquid silicone due to its durability, thermal stability, and low immunogenicity [9]. The pulmonary effects of fluid silicone are secondary to microembolization, proven by the presence of silicone globules in pulmonary macrophages and lung capillaries on histopathology [6]. Proposed mechanisms for silicone embolism syndrome include local massage into tissue, migration effect, local tissue damage resulting in access to bloodstream for embolization, and accidental injection into venous circulation [6]. Hain described a patient who was injected with corn oil for gluteal augmentation who presented with reduced consciousness, scattered petechial hemorrhages, and acute respiratory depression syndrome [10]. Alternatively, the cause of death was determined to be multiple organ failure due to systemic fat embolism. Fat embolism and silicone embolism syndrome are two potential mechanisms for the pulmonary damage in our patient as there is significant pathophysiologic overlap. As seen in this patient, both of these entities may present with hypoxemia, fever, tachycardia, and tachypnea due to obstruction of the pulmonary capillaries [9]. Additionally, complications such as obtundation and loss of neurological function have been previously described from non-facial aesthetic injections secondary to silicone embolism syndrome [11]. Although clinically significant neurological deficits were not seen in this patient, it is important to additionally monitor for any neurologic signs that may occur from microemboli traveling to the central nervous system circulation [11].

The manifestation of acute bilateral vision loss in our patient resulted from arteriolar occlusion likely due to Purtscher-like retinopathy. Purtscher-like retinopathy has multiple potential causes including fat embolism, acute pancreatitis, and renal failure. The patient demonstrated the typical clinical presentation of sudden vision loss occurring hours to days after the inciting event and presumed embolic occlusion of the pre-capillary arterioles. Ophthalmic exam revealed the characteristic cotton wool spots and retinal hemorrhages, but did not show Purtscher flecken, which is seen in 63% of patients with Purtscher’s and Purtscher-like retinopathy [12]. Purtscher flecken at the time of the acute episode remains a possibility as the patient did not present to ophthalmology clinic until 6 weeks after the injection-normalization of the retina on funduscopic examination has been previously reported in Purtscher’s retinopathy patients at 8 weeks of follow up [12]. The lack of visual improvement in this patient after 8 months of observation is not typical as the majority of patients with Purtscher-like retinopathy demonstrate variable visual recovery. However, this patient presented with retinal capillary non-perfusion, which has been shown to be a poor prognostic factor for visual recovery [13].

Although the pathogenesis underlying Purtscher’s and Purtscher-like retinopathy remains unknown, multiple mechanisms proposed include microembolization of air, fat, platelets, fibrin, or leukocyte aggregates that occlude precapillaries [13]. In addition to these, it is possible that the source of occlusion in this case was direct embolization of the injected substance. An echocardiogram was performed, ruling out a patent foramen ovale and other cardiac defects. However, this does not preclude the possibility of direct microembolization to the arterial circulation as evidenced in previously published series. Ellenbogen et al. [6] described a patient who received silicone injections for breast augmentation, after which the patient became unresponsive and subsequently died within ten hours. Post-mortem histologic examination confirmed by absorption spectrography revealed silicone deposits in the lungs, liver, brain, kidney, spleen, and pancreas. The mechanism of circumventing the pulmonary capillary circulation in the absence of cardiac defects is unclear, but embolism through recruitable arteriovenous shunts has been used to explain systemic fat globules after orthopedic procedures [14].

In summary, as the popularity of cosmetic procedures continues to grow, so will the subset of patients willing to undergo these procedures in an unregulated and precarious setting at a discounted rate. Large volume illicit injections may cause multisystem organ failure including permanent vision loss secondary to retinal vascular ischemia. This case highlights the need to raise awareness of these potentially crippling consequences to reduce the frequency of such unsafe practices.
